# Supplementary material for: Gain of Alternative Allele Expression of LINC02449 at rs149707223 in Schizophrenia and Bipolar Disorder: Inducing Synaptic Transmission and Behavioral Deficits in Mice
Source: Nat Commun. 2025 Nov 4;16:9724. doi: 10.1038/s41467-025-64717-z (PMC12586535; doi:10.1038/s41467-025-64717-z)
Supplement: Supplementary file 1 — Supplementary Information [file 41467_2025_64717_MOESM1_ESM.pdf]

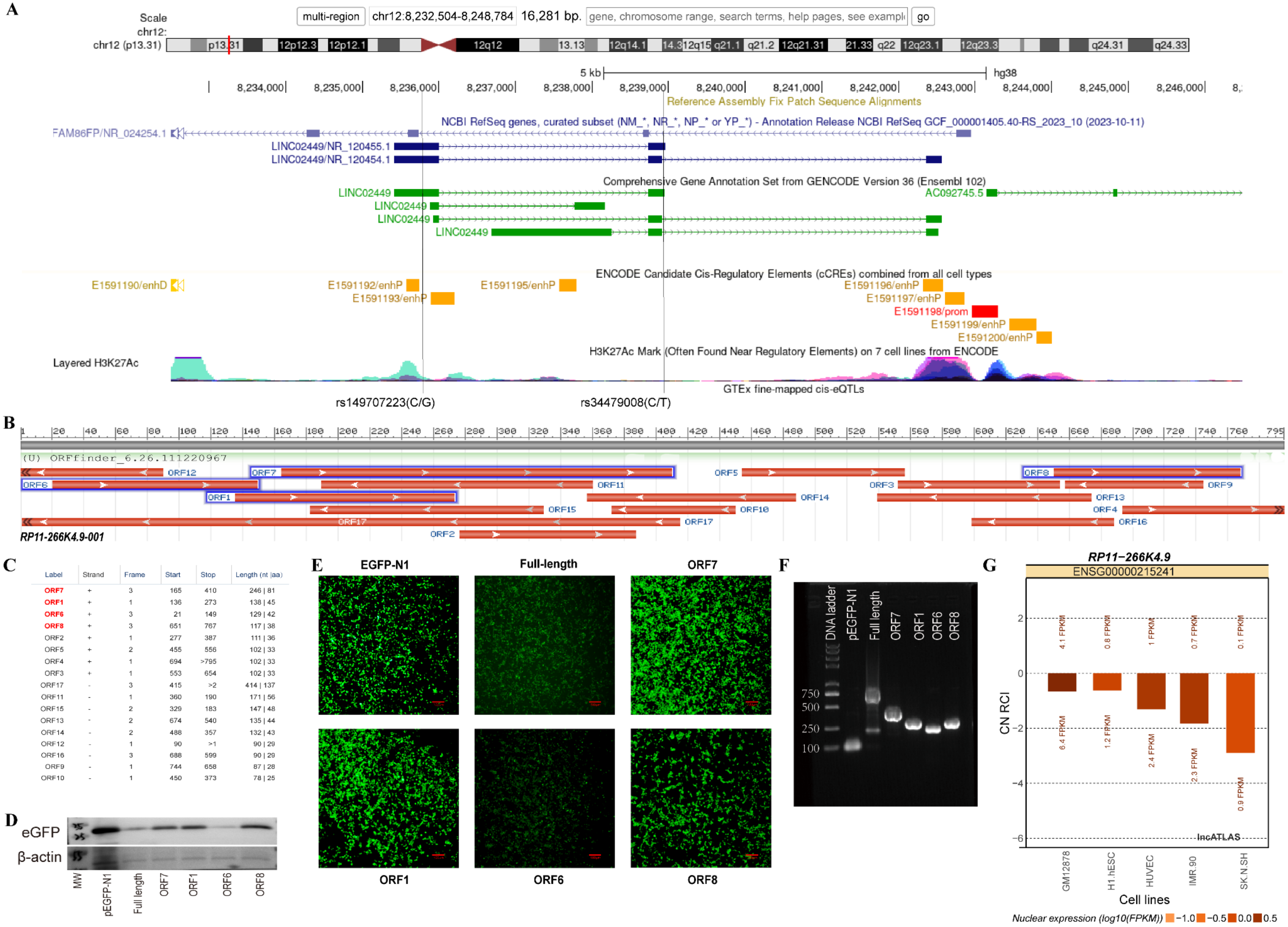

Label	Strand	Frame	Start	Stop	Length (nt |aa)
ORF7	+	3	165	410	246 | 81
ORF1	+	1	136	273	138 | 45
ORF6	+	3	21	149	129 | 42
ORF8	+	3	651	767	117 | 38
ORF2	+	1	277	387	111 | 36
ORF5	+	2	455	556	102 | 33
ORF4	+	1	694	>795	102 | 33
ORF3	+	1	553	654	102 | 33
ORF17	-	3	415	>2	414 | 137
ORF11	-	1	360	190	171 | 56
ORF15	-	2	329	183	147 | 48
ORF13	-	2	674	540	135 | 44
ORF14	-	2	488	357	132 | 43
ORF12	-	1	90	>1	90 | 29
ORF16	-	3	688	599	90 | 29
ORF9	-	1	744	658	87 | 28
ORF10	-	1	450	373	78 | 25

Supplementary Figure 1. Characterization of *LINC02449*. (**A**) Genomic annotations of *LINC02449* displayed via the UCSC Genome Browser (hg38, chr12:8,232,504–8,248,784), showing transcript isoforms (RefSeq and GENCODE), overlapping enhancers (ENCODE cCREs) and histone modification signals (H3K27Ac). (**B**) Predicted open reading frames (ORFs) within *LINC02449* as identified by NCBI ORFfinder. (**C**) Summary of the ORFs including strand, frame, and predicted peptide length based on Phylogenetic Codon Substitution Frequency (PhyloCSF) analysis, suggesting a lack of coding potential. (**D–F**) HEK293T cells transfected with full-length LINC02449 or selected ORF fused to EGFP in the pEGFP-N1 vector were analyzed by Western blotting (**D**) and immunofluorescence imaging (**E**) with EGFP antibody, or RT-PCR of the fused LINC02449–EGFP transcript (**F**). RT-PCR revealed larger products in the fused constructs compared to the empty pEGFP-N1 vector, confirming transcription of LINC02449–EGFP RNA (A non-specific product corresponding to a ~250 bp PCR fragment was observed in the full-length LINC02449 lane). Although GFP signals were detectable, Western blot bands showed similar molecular weights (MW), indicating that neither full-length LINC02449 nor its selected ORFs encode proteins. β-actin served as a loading control. (**G**) Subcellular localization of *LINC02449* based on RNA-seq data from the lncATLAS database (http://lncatlas.crg.eu). Cytoplasmic-to-nuclear (C/N) ratios are shown across various human cell lines, confirming predominant nuclear localization. The uncropped scan of the blots presented in panel D is provided at the end of the Supplementary Figure file.

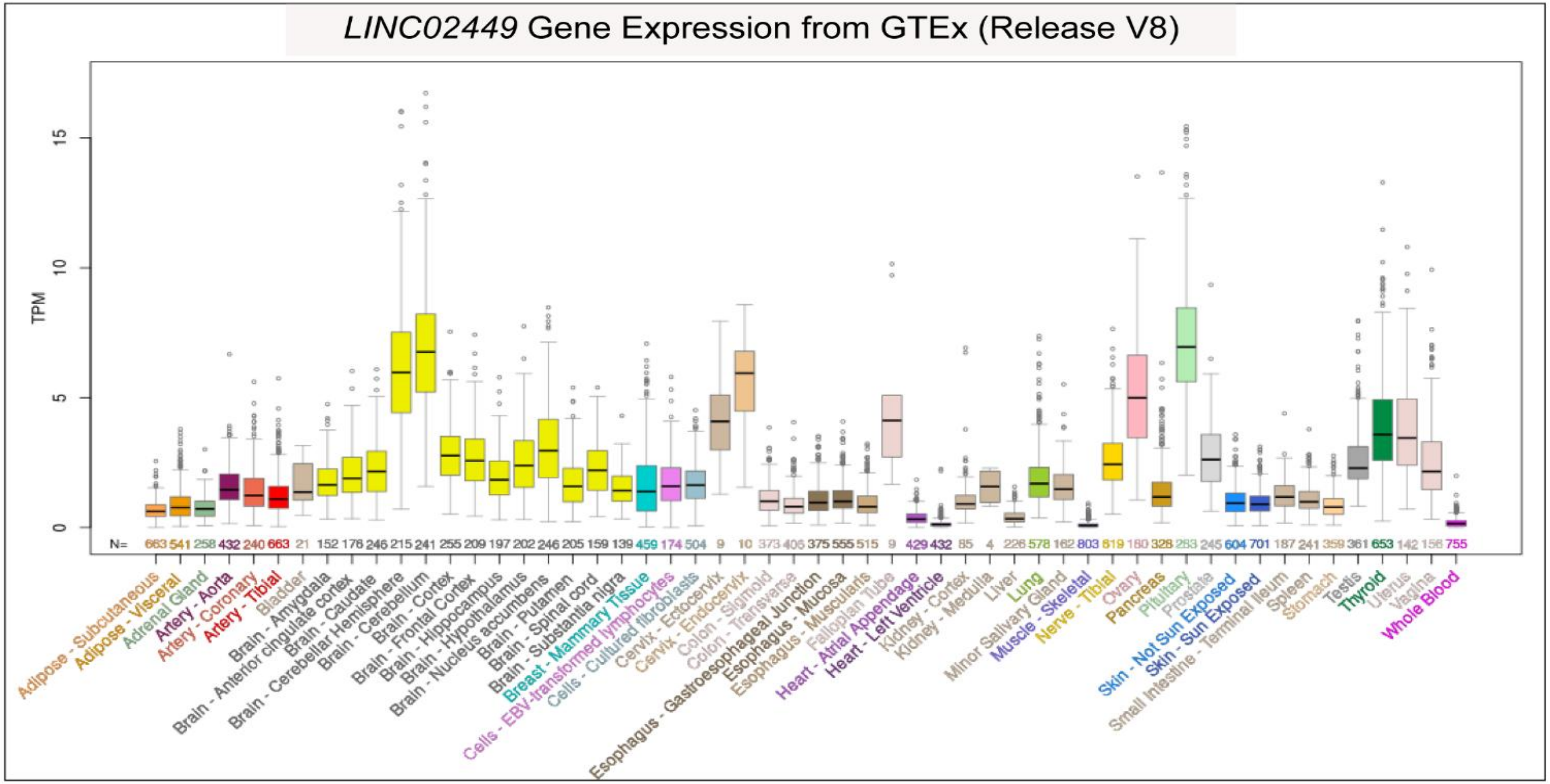

Supplementary Figure 2. Tissue expression patterns of *LINC02449* from the GTEx datasets. Boxplots depict the expression levels of *LINC02449* (in TPM) across 54 human tissues. Data were obtained from the GTEx Portal (v8), and each box represents the interquartile range with the median indicated by a horizontal line. Whiskers extend to 1.5× the interquartile range, and outliers are shown as individual dots. Notably, *LINC02449* expression is highest in brain regions, particularly the frontal cortex, anterior cingulate cortex, and hippocampus, suggesting potential roles in central nervous system function.

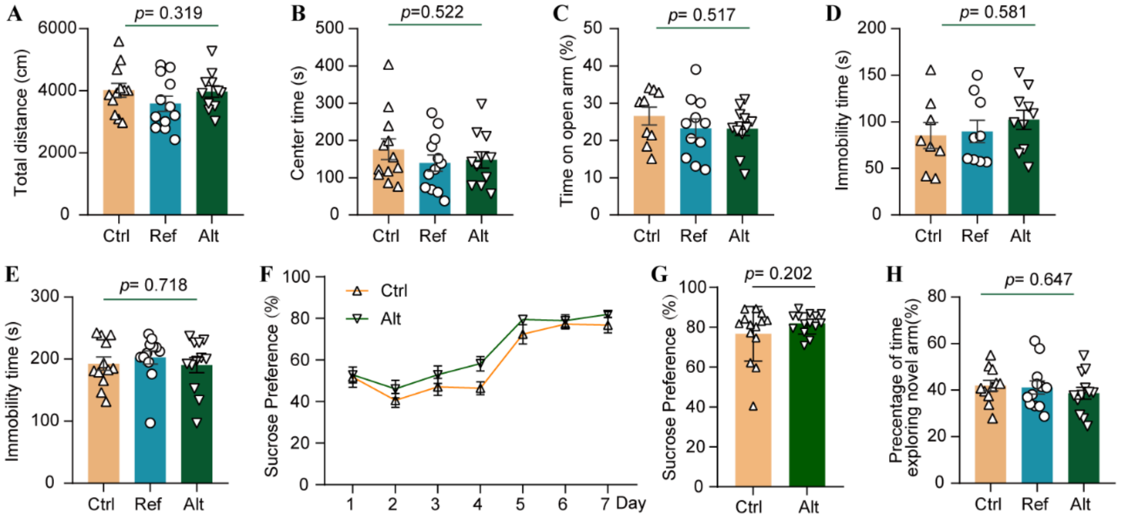

Supplementary Figure 3. Behavioral performance of control mice (Ctrl) and mice overexpressing *LINC02449* reference (Ref) or alternative (Alt) alleles. Behavioral assays include: the three-chamber social interaction test (**A, B**), elevated plus maze (**C**), forced swim test (**D**), tail suspension test (**E**), sucrose preference test from day 1 to 7 (**F**) and on day 7 (**G**), and Y-maze test (**H**). Data points for individual mice (n ⩾ 8) are shown using distinct symbol (Δ, O, ▽) and presented as mean ± SEM. Overall P values were calculated using one-way ANOVA for the indicated comparisons among the Ctrl, Ref and Alt mice (panels A-E, and H), and P values were calculated using a two-tailed Student's t-test (panel G).

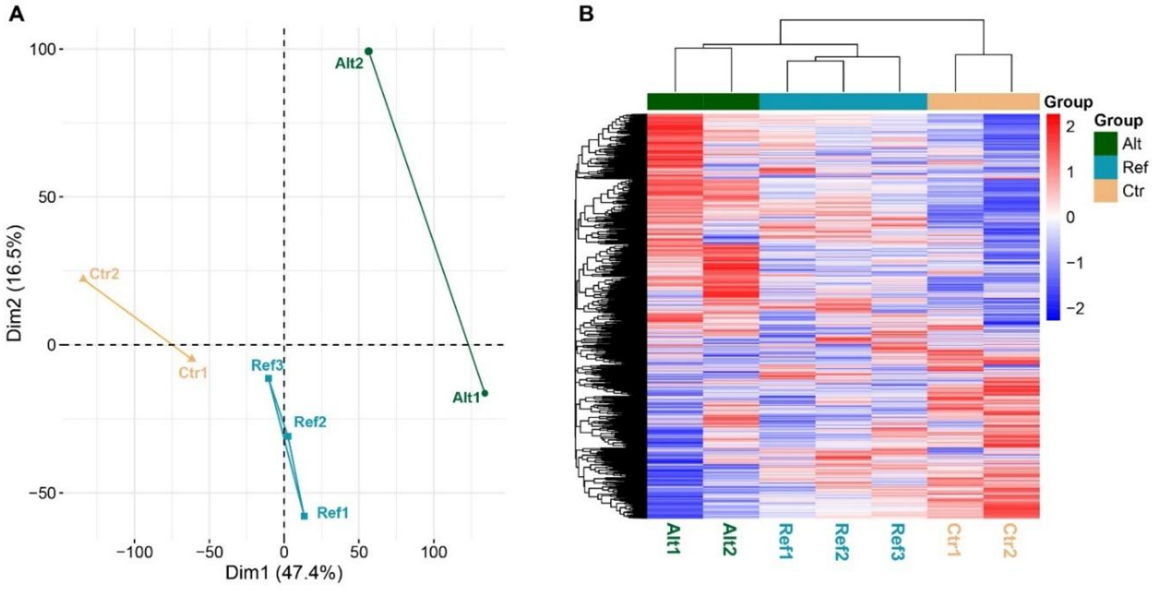

Supplementary Figure 4. Principal component analysis (PCA) and hierarchical clustering of transcriptomes from lncRNA-overexpressing mPFC. (**A**) PCA of mPFC transcriptomes from mice overexpressing the *LINC02449* reference allele (Ref), alternative allele (Alt), or control vector (Ctrl). Genes with average FPKM > 1 were included in the analysis. The first two principal components (Dim1 and Dim2) account for 63.9% of total variance. The Ref group exhibits an intermediate transcriptomic profile between Alt and Ctrl. (**B**) Hierarchical clustering heatmap of normalized gene expression (Z-scores) shows distinct clustering of Alt samples, with Ref samples positioned between Alt and Ctrl, indicating a dosage-dependent effect of the *LINC02449* allele on gene expression.

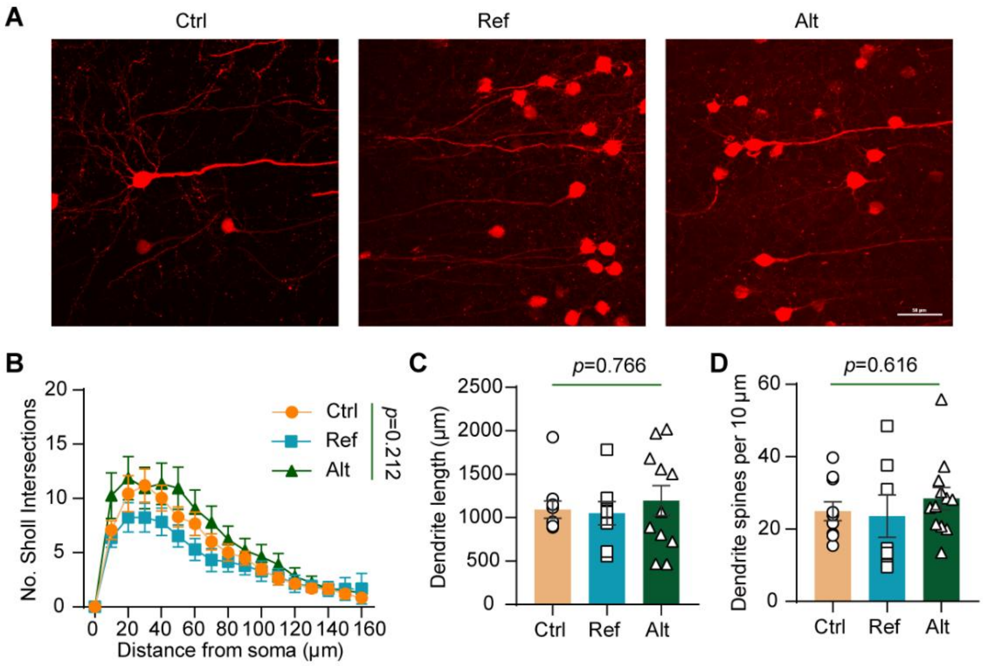

Supplementary Figure 5. Morphological analysis of mPFC pyramidal neurons in mice overexpressing LINC02449 alleles. (**A**) Representative confocal images of pyramidal neurons in the mPFC from Ctrl, Ref, and Alt mice. Neurons were fluorescently labeled by co-injection of AAV-fDIO-mCherry and AAV-CaMKII-FLP. Scale bar = 50 μm. (**B**) Sholl analysis showing the number of dendritic intersections as a function of distance from the soma. (**C**) Quantification of total dendritic length per neuron. (**D**) Quantification of dendritic spine density (spines per 10 μm). Overall P values were calculated using two-way ANOVA (panel B) or one-way ANOVA (panels C and D) for the indicated comparisons among Ctrl, Ref, and Alt mice. Data are presented as mean ± SEM. Individual data points are shown using distinct symbols.

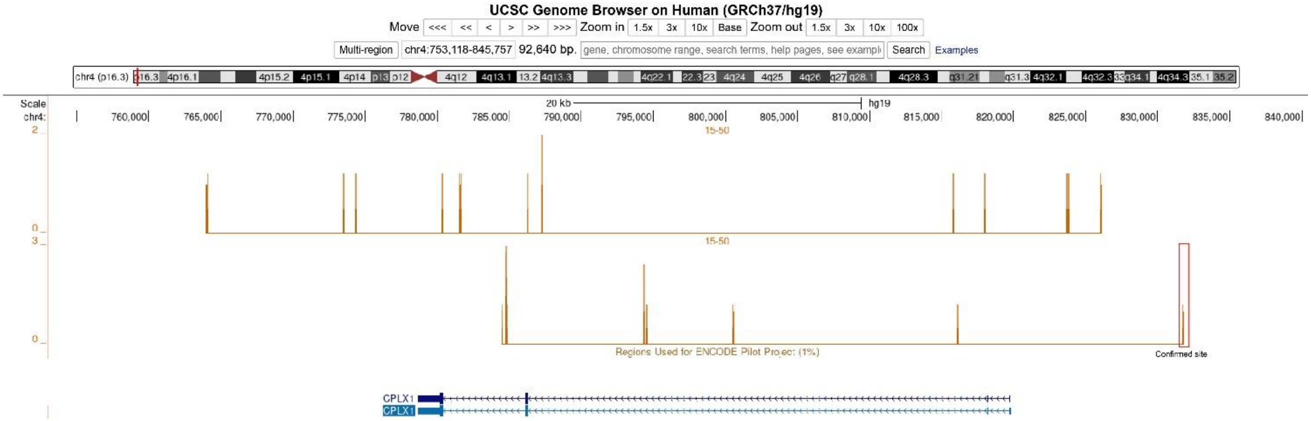

Supplementary Figure 6. Predicted *LINC02449* binding sites in the *CPLX1* upstream region. Putative RNA: DNA triplex-forming regions (orange track peaks) for *LINC02449* in the *CPLX1* gene body and upstream region were predicted using the LongTarget algorithm. A prominent binding peak located ~5,572-5,650 bp upstream of the *CPLX1* transcription start site was experimentally validated by ChIPR-qPCR. The bottom track shows the genomic structure and location of the *CPLX1* gene.

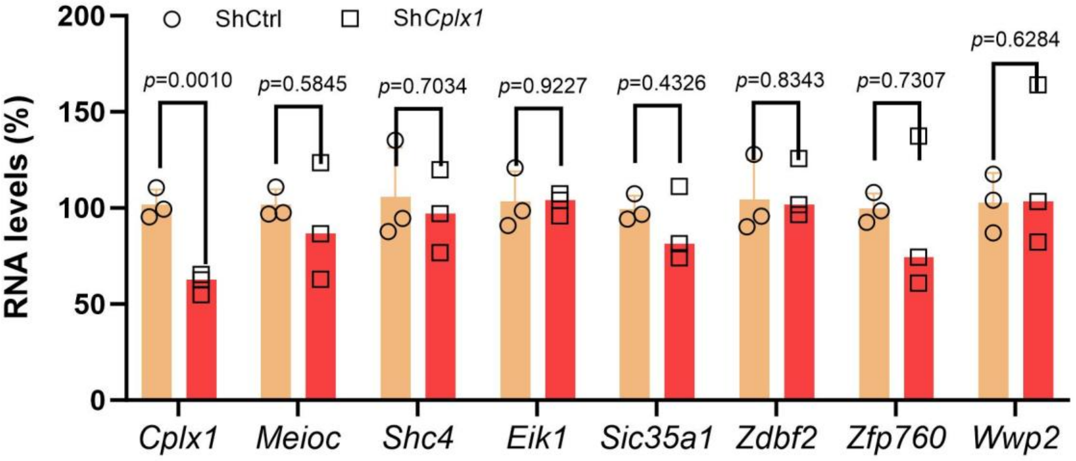

Supplementary Figure 7. **Assessment of off-target effects of Cplx1 shRNA.** Expression levels of *Cplx1* and eight top-ranked predicted off-target transcripts (*Meioc, Shc4, Eik1, Slc35a1, Zdbf2, Zfp760, Wwp2*) were measured by qPCR in mouse neuroblastoma (N2a) cells transduced with shCplx1 (red column) or control shRNA (ShCtrl, orange column). Candidate off-targets were selected based on BLAST-based sequence homology analysis. ShCtrl corresponds to a pLKO.1 vector containing a non-hairpin insert, used as a non-targeting negative control. Only *Cplx1* expression was significantly reduced following shCplx1 treatment, while no significant changes were observed in other genes. Data are presented as mean ± SEM from three independent experiments. P values were calculated using two-tailed Student's t-test.

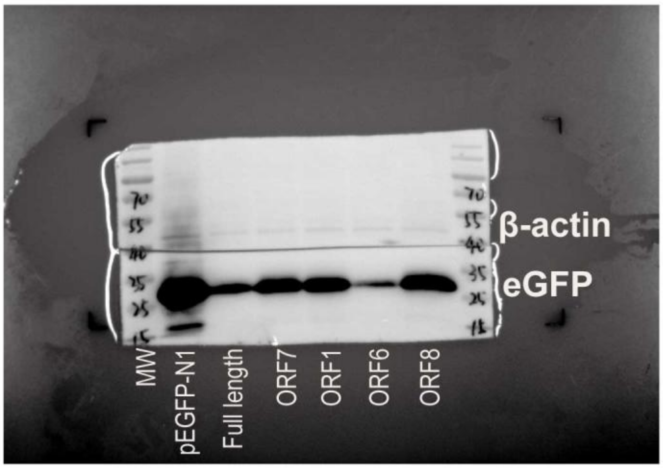

Uncropped Western blot scans corresponding to Supplementary Figure 1D. The blots show EGFP detection for full-length LINC02449 and selected ORF–EGFP fusion constructs, alongside empty vector controls. β-actin served as a loading control.
