# Supplementary material for: Gain of Alternative Allele Expression of LINC02449 at rs149707223 in Schizophrenia and Bipolar Disorder: Inducing Synaptic Transmission and Behavioral Deficits in Mice
Source: Nat Commun. 2025 Nov 4;16:9724. doi: 10.1038/s41467-025-64717-z (PMC12586535; doi:10.1038/s41467-025-64717-z)
Supplement: Supplementary file 2 — Description of Additional Supplementary Files [file 41467_2025_64717_MOESM2_ESM.pdf]

Description of Additional Supplementary Files

Supplementary Data 1. Sample information.

Supplementary Data 2. Information on ASE from twin cohorts and DEG from the PsychENCODE dataset for the 15 lncRNA ASE sites.

Supplementary Data 3. Allelic specific lncRNA binding prediction.

Supplementary Data 4. *LINC02449* genotype-dependent correlated targets (P values were calculated using linear correlation and regression analyses, with false discovery rate (FDR) adjustment).

Supplementary Data 5. DEGs induced by *LINC02449* overexpression in mouse mPFC (P values were calculated using DESeq2 analyses and adjusted using the Benjamini–Hochberg procedure (padj)).

Supplementary Data 6. DEGs induced by *LINC02449* overexpression in SK-N-SH cells (P values were calculated using DESeq2 analyses and adjusted using the Benjamini–Hochberg procedure (padj)).

Supplementary Data 7. Functional enrichment analysis of DEGs induced by *LINC02449* overexpression in SK-N-SH cells.

Supplementary Data 8. Genes co-expressed with *LINC02449* in the human prefrontal cortex (PFC) from the GTEx dataset ($r>0.3$, $p<1e-5$ were calculated using linear correlation and regression analyses).
