# Supplementary material for: Gain of Alternative Allele Expression of LINC02449 at rs149707223 in Schizophrenia and Bipolar Disorder: Inducing Synaptic Transmission and Behavioral Deficits in Mice
Source: Nat Commun. 2025 Nov 4;16:9724. doi: 10.1038/s41467-025-64717-z (PMC12586535; doi:10.1038/s41467-025-64717-z)
Supplement: Supplementary file 4 — Reporting Summary [file 41467_2025_64717_MOESM4_ESM.pdf]

nature portfolio

Corresponding author(s): Cunyou Zhao

Last updated by author(s): Aug 18, 2025

Reporting Summary

Nature Portfolio wishes to improve the reproducibility of the work that we publish. This form provides structure for consistency and transparency in reporting. For further information on Nature Portfolio policies, see our Editorial Policies and the Editorial Policy Checklist.

Statistics

For all statistical analyses, confirm that the following items are present in the figure legend, table legend, main text, or Methods section.

n/a	Confirmed	
☐	☒	The exact sample size (*n*) for each experimental group/condition, given as a discrete number and unit of measurement
☐	☒	A statement on whether measurements were taken from distinct samples or whether the same sample was measured repeatedly
☐	☒	The statistical test(s) used AND whether they are one- or two-sided<br>*Only common tests should be described solely by name; describe more complex techniques in the Methods section.*
☐	☒	A description of all covariates tested
☐	☒	A description of any assumptions or corrections, such as tests of normality and adjustment for multiple comparisons
☐	☒	A full description of the statistical parameters including central tendency (e.g. means) or other basic estimates (e.g. regression coefficient) AND variation (e.g. standard deviation) or associated estimates of uncertainty (e.g. confidence intervals)
☐	☒	For null hypothesis testing, the test statistic (e.g. *F*, *t*, *r*) with confidence intervals, effect sizes, degrees of freedom and *P* value noted<br>*Give P values as exact values whenever suitable.*
☒	☐	For Bayesian analysis, information on the choice of priors and Markov chain Monte Carlo settings
☒	☐	For hierarchical and complex designs, identification of the appropriate level for tests and full reporting of outcomes
☐	☒	Estimates of effect sizes (e.g. Cohen's *d*, Pearson's *r*), indicating how they were calculated

Our web collection on statistics for biologists contains articles on many of the points above.

Software and code

Policy information about availability of computer code

Data collection	The software utilized in this study is described within the manuscript.
Data analysis	This research does not involve any custom algorithms or software.

For manuscripts utilizing custom algorithms or software that are central to the research but not yet described in published literature, software must be made available to editors and reviewers. We strongly encourage code deposition in a community repository (e.g. GitHub). See the Nature Portfolio guidelines for submitting code & software for further information.

Data

Policy information about availability of data

All manuscripts must include a data availability statement. This statement should provide the following information, where applicable:

- Accession codes, unique identifiers, or web links for publicly available datasets
- A description of any restrictions on data availability
- For clinical datasets or third party data, please ensure that the statement adheres to our policy

The RNA-seq data generated in this study (including quantification and raw sequencing files) have been deposited in the GEO database under the accession codes GSE295729 (for SK-N-SH cells; https://www.ncbi.nlm.nih.gov/geo/query/acc.cgi?acc=GSE296023), and GSE296023 (for murine brain tissues; https://www.ncbi.nlm.nih.gov/geo/query/acc.cgi?acc=GSE296023). Source data are provided with this paper. All data supporting the findings described in this manuscript are available in the article and in the Supplementary Information.

Research involving human participants, their data, or biological material

Policy information about studies with human participants or human data. See also policy information about sex, gender (identity/presentation), and sexual orientation and race, ethnicity and racism.

Reporting on sex and gender	Gender-based analyses were not performed.
Reporting on race, ethnicity, or other socially relevant groupings	No race- and ethnicity- based analysis have been performed.
Population characteristics	See above.
Recruitment	In this study, we recruited nine pairs of MZ twins discordant affected by psychiatric disorders (PDC), including four pairs discordant for SZ (SDC) and five pairs discordant for BD (BDC), as detailed in Supplementary Table 1. Zygosity was determined using the Qiagen Investigator Argus X-12 QS Kit (Qiagen, USA). All participants met the diagnostic criteria for SZ or BD as defined in the fifth edition of the Diagnostic and Statistical Manual of Mental Disorders (DSM-V). Prior to participation, all individuals provided written informed consent after receiving a comprehensive explanation of the study procedures.
Ethics oversight	The study was approved by the Medical Ethics Committee of Zhujiang Hospital of Southern Medical University (#2022-KY-086), Guangdong Provincial People's Hospital (#KY2024-915-02), and the third People's Hospital of Zhongshan (SSYLL20210301) and conducted in accordance with the Declaration of Helsinki.

Note that full information on the approval of the study protocol must also be provided in the manuscript.

Field-specific reporting

Please select the one below that is the best fit for your research. If you are not sure, read the appropriate sections before making your selection.

☒ Life sciences ☐ Behavioural & social sciences ☐ Ecological, evolutionary & environmental sciences

For a reference copy of the document with all sections, see nature.com/documents/nr-reporting-summary-flat.pdf

Life sciences study design

All studies must disclose on these points even when the disclosure is negative.

Sample size	No statistical methods were used to pre-determine sample sizes.
Data exclusions	In all experiments, mice with infected/bleeding/unhealthy conditions after surgery were excluded from behavioural testing, and mice with missing viral injections or implantation targets as depicted in the brain atlas were not included in the experimental analyses.
Replication	Behavioural experiments were repeated multiple times on independent mice, with at least two individuals independently analyzing the time points of behavioural events. Measurements were taken on individual mice/cells from multiple biological replicates. All are described in Methods and Results. The number of replicates (n) is shown in the legend.
Randomization	All investigators were unaware of subgroup assignments during the data collection and analysis process.
Blinding	All investigators were unaware of subgroup assignments during the data collection and analysis process.

Reporting for specific materials, systems and methods

We require information from authors about some types of materials, experimental systems and methods used in many studies. Here, indicate whether each material, system or method listed is relevant to your study. If you are not sure if a list item applies to your research, read the appropriate section before selecting a response.

Materials & experimental systems

n/a	Involved in the study
☐	☒ Antibodies
☐	☒ Eukaryotic cell lines
☒	☐ Palaeontology and archaeology
☐	☒ Animals and other organisms
☒	☐ Clinical data
☒	☐ Dual use research of concern
☒	☐ Plants

Methods

n/a	Involved in the study
☒	☐ ChIP-seq
☒	☐ Flow cytometry
☒	☐ MRI-based neuroimaging

Antibodies

Antibodies used	For western blot assays: (CPLX1, Proteintech, 1:5000; GAPDH, Proteintech, 1:10000. Horseradish peroxidase (HRP)-conjugated goat-anti-rabbit, Proteintech, 1:5000).
Validation	CPLX1, Proteintech, https://www.ptgcn.com/products/CPLX1-Antibody-10246-2-AP.htm.<br>GAPDH, Proteintech, https://www.ptgcn.com/products/GAPDH-Antibody-60004-1-Ig.htm.<br>HRP-conjugated Goat Anti-Rabbit IgG(H+L), Proteintech, https://www.ptgcn.com/products/HRP-conjugated-Affinipure-Goat-Anti-Rabbit-IgG-H-L-secondary-antibody.htm.<br>These antibodies has been validated in multiple publications.

Eukaryotic cell lines

Policy information about cell lines and Sex and Gender in Research

Cell line source(s)	Human neuroblastoma SK-N-SH (HTB-11) and human neuroblastoma SH-SY5Y (CRL-2266) cell lines were originally obtained from the ATCC. Product category: Human cells. Organism: Homo sapiens, human. Morphology: epithelial. Tissue: brain. Product format: Frozen. Storage conditions: Vapor phase of liquid nitrogen.<br>Mouse neuroblastoma Neuro-2a (CCL-131) cell lines were originally obtained from the ATCC. Product category: Mouse cells. Organism: Mus musculus, mouse. Morphology: neuroblast. Tissue: brain. Product format: Frozen. Storage conditions: Vapor phase of liquid nitrogen.<br>HEK293T (CRL-11268) cell lines were originally obtained from the ATCC. Product category: Human cells. Organism: Homo sapiens, human. Morphology: epithelial. Tissue: kidney. Product format: Frozen. Storage conditions: Vapor phase of liquid nitrogen.
Authentication	All cell lines were obtained from ATCC and authenticated by this organization with certificates.
Mycoplasma contamination	We confirm that all cell lines were negative for mycoplasma contamination.
Commonly misidentified lines<br>(See ICLAC register)	No commonly misidentified cell lines were used in this study.

Animals and other research organisms

Policy information about studies involving animals; ARRIVE guidelines recommended for reporting animal research, and Sex and Gender in Research

Laboratory animals	The experiments were conducted using male C57BL/6 mice, aged 6–8 weeks, obtained from the Laboratory Animal Center, Southern Medical University. Four to five C57BL/6J mice were housed in an EVC cage (300×170×120 mm) at 23±1 °C, humidity 40% under standard laboratory conditions with a 12 h light/dark cycle (lights on from 8:00 a.m. to 8:00 p.m.) and with free access to food and water.
Wild animals	No wild animals were used in this study.
Reporting on sex	All studies employed male mice.
Field-collected samples	No field-collected samples were used in this study.
Ethics oversight	All of the experiments were conducted in accordance with the Regulations for the Administration of Affairs Concerning Experimental Animals (China) and were approved by the Institutional Animal Care and Use Committee and the Ethics Committee of Guangdong General Hospital of Southern Medical University (#KY2024-915-02).

Note that full information on the approval of the study protocol must also be provided in the manuscript.

Plants

Seed stocks	*Report on the source of all seed stocks or other plant material used. If applicable, state the seed stock centre and catalogue number. If plant specimens were collected from the field, describe the collection location, date and sampling procedures.*
Novel plant genotypes	*Describe the methods by which all novel plant genotypes were produced. This includes those generated by transgenic approaches, gene editing, chemical/radiation-based mutagenesis and hybridization. For transgenic lines, describe the transformation method, the number of independent lines analyzed and the generation upon which experiments were performed. For gene-edited lines, describe the editor used, the endogenous sequence targeted for editing, the targeting guide RNA sequence (if applicable) and how the editor was applied.*
Authentication	*Describe any authentication procedures for each seed stock used or novel genotype generated. Describe any experiments used to assess the effect of a mutation and, where applicable, how potential secondary effects (e.g. second site T-DNA insertions, mosiacism, off-target gene editing) were examined.*
